# Adjusting for covariates on a slippery slope: linkage analysis of change over time

**DOI:** 10.1186/1471-2156-4-S1-S50

**Published:** 2003-12-31

**Authors:** Evadnie Rampersaud, Andrew Allen, Yi-Ju Li, Yujun Shao, Meredyth Bass, Carol Haynes, Allison Ashley-Koch, Eden R Martin, Silke Schmidt, Elizabeth R Hauser

**Affiliations:** 1 Section of Medical Genetics, Department of Medicine, Center for Human Genetics, Duke University Medical Center, Durham, North Carolina, USA; 2 Department of Biostatistics and Bioinformatics, Duke University, Durham, North Carolina, USA

## Abstract

**Background:**

We analyzed the Genetic Analysis Workshop 13 (GAW13) simulated data to contrast and compare different methods for the genetic linkage analysis of hypertension and change in blood pressure over time. We also examined methods for incorporating covariates into the linkage analysis. We used methods for quantitative trait loci (QTL) linkage analysis with and without covariates and affected sib-pair (ASP) analysis of hypertension followed by ordered subset analysis (OSA), using variables associated with change in blood pressure over time.

**Results:**

Four of the five baseline genes and one of the three slope genes were not detected by any method using conventional criteria. OSA detected baseline gene b35 on chromosome 13 when using the slope in blood pressure to adjust for change over time. Slope gene s10 was detected by the ASP analysis and slope gene s11 was detected by QTL linkage analysis as well as by OSA analysis. Analysis of null chromosomes, i.e., chromosomes without genes, did not reveal significant increases in type I error. However, there were a number of genes indirectly related to blood pressure detected by a variety of methods.

**Conclusions:**

We noted that there is no obvious first choice of analysis software for analyzing a complicated model, such as the one underlying the GAW13 simulated data. Inclusion of covariates and longitudinal data can improve localization of genes for complex traits but it is not always clear how best to do this. It remains a worthwhile task to apply several different approaches since one method is not always the best.

## Background

In our analysis of the Genetic Analysis Workshop 13 (GAW13) simulated data for Replicate 1 with no missing data we had two goals: 1) use the longitudinal information to improve our linkage analysis and 2) incorporate covariates in our linkage analysis. At present, there are no standard tools for linkage analysis of longitudinal data. In addition, the incorporation of covariates in linkage analysis is a very active area of current methodological development. As a result there was no "industry standard" analysis for either of our goals. Consequently, we explored several approaches to detect the simulated genes. We opened the answers at the beginning and used the answers extensively to evaluate our approach.

We investigated different approaches for handling data from repeated measurements, in this case multiple phenotype values collected at different time points. We used subject-specific slope values to represent change in systolic blood pressure (SBP) over time and considered the effects of adjusting slopes for potentially important covariates before the linkage analysis. We used these slopes directly as traits in quantitative analysis, and as covariates in linkage analysis of the hypertension as a binary affection status variable. We chose change over time in SBP as the quantitative trait for SOLAR multipoint linkage analysis, and used the presence of hypertension (HBP) to define a binary affection status for affected sib-pair (ASP) multipoint linkage analysis with SIBLINK [2]. A recently developed program for ordered subset analysis (OSA) [3] was used in conjunction with SIBLINK to examine whether phenotypically homogeneous subgroups of families, based on family-specific mean levels of the slope covariates, gave improved linkage signals for the underlying simulated SBP quantitative trait loci (QTL), particularly those involved in determining the rate of change in BP over time.

A key issue in our investigation involved determining which covariates to use and at what point in our analyses to adjust for them. We were interested in examining how inclusion of all covariates in a general model and subsequent covariate elimination using a variance-component analysis method, SOLAR, compared with analysis with one covariate at a time using a method like OSA, in conjunction with ASP linkage analysis. A related question was whether covariates should be included at a family or individual level. With OSA, the mean covariate values are taken over all time points for each individual and then averaged at the family level. We examined how this approach might differ from the inclusion of individual means for all covariates in a mixed model using a variance-components approach.

We used only one replicate in our analysis. Our initial goals were implementation of the analysis plan and a limited consideration of whether our analysis methods could detect any of the genes involved in change in blood pressure over time. An analysis of all replicates and further simulation studies are required to begin to make statements about the general performance of covariate-adjusted linkage methods.

## Methods

We used the GAW13 simulated complete data set for Replicate 1 consisting of 2860 genotyped individuals in 330 families. The data set was examined using three genetic analysis programs: SIBLINK [2], Ordered Subset Analysis (OSA) [3], and Sequential Oligogenic Linkage Analysis Routines (SOLAR) [1]. We chose to analyze chromosomes 5, 7, 13, 15, and 21 because they contained genes that determine blood pressure, acting either at baseline or in change over time. We analyzed four null chromosomes (2, 4, 6, 10) to get a sense of the number of type I errors using OSA based on the mean covariate values in affected/unaffected individuals. We found that the false positive rate was consistent with the nominal significance level (data not shown).

Subject-specific slopes reflecting individual change over time of SBP were computed with SAS-PROC MIXED [4] using three different models: unadjusted slope, adjusted slope, and the "true" slope. The unadjusted slopes fit a line to the observed longitudinal BP values. The adjusted slopes fit a model including individual environmental variables that we considered important in the absence of knowledge about the true simulation model. Fixed effects were fit for age, gender, smoking, drinking, and cohort, and both fixed and random effects were fit for hypertension treatment. We also computed slope values that were as close as possible to the "true" values by using the simulating model for SBP provided in the GAW13 answers, including the correct transformations of the involved variables. To account for the different number of time points contributing to the slope estimates for each subject, we calculated normalized slope values (slope divided by standard deviation of the estimate).

Variance components analysis (SOLAR) was used to perform multipoint linkage analysis using the normalized individual slope values for SBP (unadjusted, adjusted, and true) as the quantitative trait values. All families were included in this analysis. A common constant was added to make all estimated slope values positive, then the data were log-transformed and outliers were eliminated to approximate a normal trait distribution. Heritability was estimated from the best-fitting polygenic model, starting with a model that included age (at onset of HBP for affected individuals, at exam for unaffecteds) and average values (over all time points) of cholesterol, glucose, HDL, triglycerides, height, weight, and body mass index (BMI). Once this model was generated, multipoint linkage analysis using a variance components approach was carried out using SOLAR.

SIBLINK v. 3.0 was used to perform multipoint ASP linkage analysis using HBP as indicated in the data set as a dichotomous disease trait. There were 171 families with at least one ASP included in our SIBLINK analysis, for a total of 575 ASPs with hypertension. SIBLINK was used to calculate family-specific multipoint LOD scores across each chromosome, based on estimated identity by descent (IBD) status among affected sibling pairs. The OSA program then utilized the multipoint LOD scores and covariate values from each family, attempting to identify homogeneous subsets of families presenting increased evidence for linkage. In theory, examining the data from a homogeneous subset of linked families yields a more accurate estimate of disease gene location because the location estimate is not influenced by unlinked families. The OSA program takes as input, any multipoint set of additive, family-specific linkage scores, such as LOD scores from SIBLINK or nonparametric LOD scores from GENEHUNTER Plus (Kong and Cox). The OSA program ranked families by mean values of the covariates: the calculated slope values for SBP and additional covariates age (age at onset of HBP for affected individuals, age at exam for unaffecteds), cholesterol, glucose, HDL, triglycerides, height, weight, and BMI. Family-specific means were calculated i) for all affected individuals and ii) for all family members regardless of affection status (individual values were averages over all time points). Using one covariate at a time, family-specific multipoint LOD scores were added in the covariate-based rank order and the maximum LOD score for any subset of families was determined for that covariate. The significance of the increase in the subset-based LOD score over the global LOD score from all families was assessed with an empirical *p*-value based on randomly permuting the order in which families were added.

## Results

The linkage results for slope variables compared with the known locations of simulated SBP genes for chromosomes 5, 7, 13, 15, and 21 are shown in Table 1 (SOLAR results) and Table 2 (ASP linkage and OSA results). Results are presented for OSA using family-specific means for affected individuals as well as family-specific means including all individuals in the family. We found no consistent improvement in the linkage signal when using the mean of all pedigree members compared with the mean of affected members only, however there could be considerable differences when using the two sets of means. Tables 1 and 2 also show the results when using the normalized slopes and for the non-normalized slopes. In most cases the non-normalized results gave stronger results.

**Figure 1:**
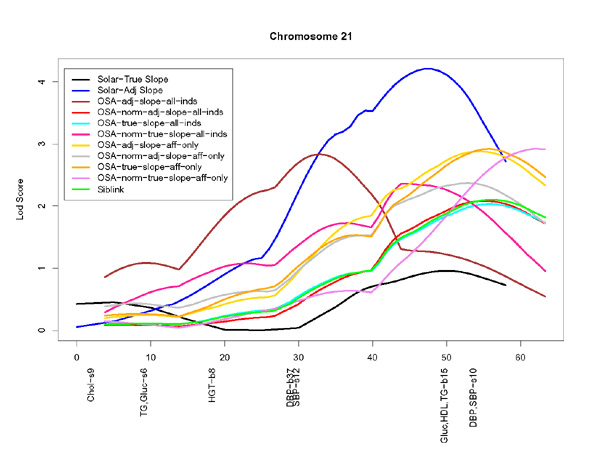
**LOD score curves for linkage results on chromosome 21. **The x-axis is in cM along with the location of genes on that chromosome. SOLAR curves are for the normalized SBP slopes. The SIBLINK curve shows the ASP LOD score for HBP.

Using standard criteria for identifying regions of interest (SOLAR LOD > 1.5, ASP LOD > 1.0, or OSA *p*-value < 0.05), we did not observe any linkage signals within 20 cM of any baseline SBP genes on chromosomes 5, 7, or 13 with ASP combined with ordered subsets analyses (SIBLINK/OSA) using the mean of normalized slope values for all family members or using variance component analysis on the calculated normalized SBP slope values. OSA was able to identify gene s11 on chromosome 15 (LOD 2.81 at position 5.1 cM, using mean of unadjusted slope over affected family members only). Not surprisingly, since s10 exerts a relatively strong effect on the variance of SBP over time, our methods were most successful at identifying slope gene s10 on chromosome 21 (Figure 1). Analysis of the dichotomized trait (HBP) with SIBLINK led to the detection of s10 on chromosome 21 (LOD 2.03 at position 56 cM). OSA did not provide a significant improvement of this score. We also found significant results with variance component analysis (SOLAR) using the adjusted SBP slope and the true slope values, both of which localized within 4 cM of s10 at 54 cM on chromosome 21. Aside from this result, SOLAR did not detect any other slope genes despite the fact that estimated heritabilities were significant (normalized slope H^2 ^= 18.10%; normalized adjusted slope H^2 ^= 28.4%; normalized true slope H^2 ^= 24%). Interestingly, non-normalized slope values used in SOLAR tended to have higher heritability estimates than normalized slopes (slope H^2 ^= 29.7%; adjusted slope H^2 ^= 44.5%; true slope H^2 ^= 34.20%), although the increase in the heritabilities did not automatically lead to larger LOD scores. The analysis of non-normalized adjusted slope (H^2 ^= 44.5%) found s11 on chromosome 15 with LOD = 1.59 and s10 on chromosome 21 with LOD = 6.80. We analyzed Replicate 7 of the simulated data and confirmed that the non-normalized values do appear to give stronger results than the normalized values in that replicate as well.

**Figure 2:**
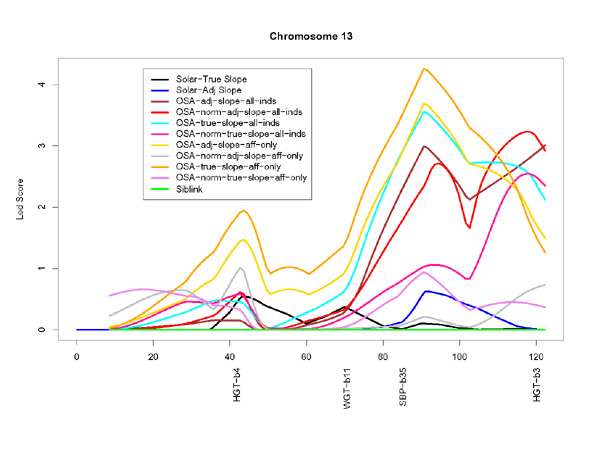
**LOD score curves for linkage results on chromosome 13. **The x-axis is in cM along with the location of genes on that chromosome. SOLAR curves are for the normalized SBP slopes. The SIBLINK curve shows the ASP LOD score for HBP.

We also observed linkage signals in regions that did not harbor SBP genes. On chromosome 7 both the ordered-subset analysis and the variance-component analysis detected gene b10 (at position 124 cM, chromosome 7). In addition, ordered subset analysis detected b3 (at position 120 cM on chromosome 13, Figure 2). Both genes influence SBP indirectly through their influence on height. However, finding a direct connection between these genes, hypertension, SBP slope, and height was difficult.

## Conclusions

We noted that there is no obvious first choice of software for longitudinal data and incorporating covariates for analyzing such a complicated model as the one underlying the GAW13 simulated Replicate 1 data set. Having the answers allowed us to set up analyses to get the best possible results. However, even with the answers, the results obtained with adjustment for the true model were not superior to adjustment for generally accepted covariates.

QTL analysis using the SOLAR program had difficulty finding the true slope genes, even when the simulating model was used to calculate individual-specific slopes. Perhaps our mixed model did not capture the longitudinal aspect of these data as well as we had hoped. We realize that our approach to the longitudinal aspect of the data was rather ad hoc, stemming from the lack of longitudinal QTL software. Perhaps the slope variables we utilized in our analyses were not optimal. Weighting the likelihood contributions by the precision of the slope estimates could possibly have improved our analysis. However, the normalization procedure we used seemed to reduce the evidence for linkage in some cases, suggesting that perhaps we were removing some of the genetic variability when we normalized the slope values. As expected, we found that dichotomizing and performing ASP analysis (i.e., analyzing only the extreme tail of the distribution) can lead to successful localization if the QTL is bi-allelic, has a relatively strong effect on the mean of the distribution, and if the genotype-specific means are close to an additive model, which is assumed in ASP analysis using SIBLINK. Incidentally, this was also the situation where variance component analysis using SOLAR performed best (chromosome 21).

OSA appears to be useful in localization of complex trait genes, but it can also produce peaks for genes indirectly related to the trait of interest, particularly when there are multiple genes on the same chromosome that are correlated with the analyzed trait in a complex way. It is possible that our detection of the height genes is due to high heritability of height or strong correlations with other variables. The answers reveal that height is indirectly related to SBP through its direct contributions to other variables. When concentrating on a single maximum for the gene location, we did not detect any baseline genes for SBP with our approach. However, when there is more than one gene on a chromosome, localization of any single gene may be more difficult. For example, on chromosome 13, a high LOD score of 3.2 obtained from OSA occurs around 118 cM (near the height gene b3 at 120 cM), and 33 cM away from the SBP baseline gene b35 at 85 cM. The *p*-values for the increase in the OSA LOD scores in this region are below 0.05. We also analyzed simulated Replicate 7 to see if any of the patterns observed in Replicate 1 were observed in another replicate. Qualitatively the localization results were similar in that other genes were detected, however, we did not observe the same genes as those in Replicate 1.

There is considerable methodological work to be done to provide insight into how proximity of other genes may influence detection and localization of any individual gene. We were pleasantly surprised that we were able to obtain results for any of the baseline genes, since our analysis was weighted toward detecting slope genes. The OSA results for chromosome 13 (Figure 2) suggest that in the ASP analysis of HBP, accounting for BP change over time, may have uncovered a subset of families segregating baseline genes that were not detectable because of the variability over time. Finally, we found that, even with the answers, it is difficult to determine which covariates to use and at what stage we should adjust for these covariates that are contributing to the phenotype of interest. It is not clear that using a multivariate regression model with SOLAR is preferable to examination of covariates one-at-a-time at the modeling stage for the phenotype using OSA. It is difficult to make clear recommendations since neither software package produced consistent results pointing to the localization of SBP genes, although OSA found s11 on chromosome 15 and variance components analysis using SOLAR and ASP analysis using SIBLINK found s10 on chromosome 21. We focused our investigation on Replicate 1 of the simulated data. Clearly, extending our analyses to include all of the simulation replicates might shed more light on which methods perform better, and in which situations.

We are able to conclude from our analyses that inclusion of covariates and longitudinal data can improve localization of genes for complex traits but it is not always clear how best to do this. The information used by each method is slightly different and thus it is not at all surprising that the different methods pick up different genes in this complex system. Thus we conclude that it remains a worthwhile task to apply several different approaches since one method is not always the best.

## Figures and Tables

**Table 1:** Linkage detection on 5 chromosomes with SBP genes using SOLAR QTL variance components linkage analysis.^A^

**Analysis**	**Chr 5**	**Chr 7**	**Chr 13**	**Chr 15**	**Chr 21**
Norm^B ^SBP slope (H^2 ^= 0.18)	0.50 (140)	0.75 (102)	0.20 (70)	0.66 (15)	1.0 (56)
Norm adjusted SBP slope (H^2 ^= 0.28)	0.29 (190)	1.72 (97)	0.62 (91)	0.47 (0)	4.2 (51)
Norm "true" SBP slope (H^2 ^= 0.24)	0.40 (140)	0.74 (32)	0.54 (44)	0.89 (15)	0.96 (50)
SBP slope (H^2 ^= 0.29)	0.70 (142)	2.17 (125)	0.53 (91)	1.49 (16)	0.32 (55)
Adj SBP slope (H^2 ^= 0.44)	0.42 (55)	2.20(121)	0.52 (119)	1.59 (5)	6.8 (46)
"True" SBP slope (H^2 ^= 0.34)	0.64 (141)	1.36 (31)	1.29 (44)	1.13 (15)	0.61 (51)
True location of SBP genes (cM)	B34 (176)	B36 (47)	B35 (85)	S11 (4.5)	S12 (29) S10 (54)

^A^Values in the table are the maximum LOD score and the location in cM. ^B^Norm, normalized.

**Table 2:** Linkage detection on 5 chromosomes with SBP genes using ASP linkage analysis and OSA.^A^

**Analysis**	**Chr 5**	**Chr 7**	**Chr 13**	**Chr 15**	**Chr 21**
SIBLINK ASP on HBP OSA	0.52 (63)	0.16 (199)	0.0 (--)	0.92 (109)	2.03 (56)
Norm^B ^SBP slope (affecteds only)	1.87 (58) *p *= 0.45	*p *= 0.003	0.88 (28) *p *= 0.04	2.81 (5) *p *= 0.04	3.28 (63) *p *= 0.16
Norm SBP slope (all individuals)	1.99 (39) *p *= 0.27	3.13 (124) *p *= 0.01	2.14(117) *p *= 0.04	3.61 (99) *p *= 0.009	2.10 (56) *p *= 0.80
SBP slope (affecteds only)	2.24(127) *p *= 0.17	*p *= 0.12	3.88 (91) *p *= 0.005	2.53 (105) *p *= 0.094	2.57 (53) *p *= 0.58
SBP slope (all individuals)	2.12(112) *p *= 0.27	2.91 (12) *p *= 0.03	3.29 (91) *p *= 0.01	2.36 (106) *p *= 0.13	2.91 (57) *p *= 0.38
Norm adjusted SBP slope (affecteds only)	1.25(63) *p *= 0.66	2.07 (140) *p *= 0.13	1.01 (43) *p *= 0.44	1.87 (109) *p *= 0.23	2.37 (53) *p *= 0.62
Norm adjusted SBP slope (all individuals)	1.84(214) *p *= 0.39	2.50 (107) *p *= 0.05	3.23(118) *p *= 0.02	2.11 (99) *p *= 0.14	2.75 (53) *p *= 0.44
Adjusted SBP slope (affecteds only)	2.41(112) *p *= 0.17	1.16 (123) *p *= 0.58	3.69 (91) *p *= 0.009	1.74 (108) *p *= 0.38	2.88 (55) *p *= 0.27
Adjusted SBP slope (all individuals)	2.12(112) *p *= 0.29	2.08 (99) *p *= 0.05	3.01(122) *p *= 0.05	1.97 (107) *p *= 0.19	2.91 (53) *p *= 0.32
Norm "true" SBP slope (affecteds only)	1.34 (61) *p *= 0.74	2.08 (123) *p *= 0.15	0.94 (91) *p *= 0.41	1.96 (108) *p *= 0.23	2.92 (62) *p *= 0.45
Norm "true" SBP slope (all individuals)	2.14(214) *p *= 0.25	2.44 (124) *p *= 0.06	2.54(117) *p *= 0.05	2.20 (106) *p *= 0.14	2.72(52) *p *= 0.44
"True" SBP slope (affecteds only)	2.12(112) *p *= 0.19	1.95 (37) *p *= 0.16	4.26 (91) *p *= 0.002	1.95 (108) *p *= 0.23	2.92 (55) *p *= 0.34
"True" SBP slope (all individuals)	2.16(209) *p *= 0.24	2.37 (99) *p *= 0.07	3.56 (91) *p *= 0.009	1.89 (108) *p *= 0.24	3.15 (58) *p *= 0.17
True location of SBP genes (cM)	B34 (176)	B36 (47)	B35 (85)	S11 (4.5)	S12 (29) S10 (54)

^A^Values in the table are the maximum LOD score and the location in cM. ^B^Norm, normalized.
